# Development of a conductive biocomposite combining graphene and amniotic membrane for replacement of the neuronal network of tissue-engineered urinary bladder

**DOI:** 10.1038/s41598-020-62197-3

**Published:** 2020-04-02

**Authors:** J. Adamowicz, I. Pasternak, T. Kloskowski, M. Gniadek, S. V. Van Breda, M. Buhl, D. Balcerczyk, M. Gagat, D. Grzanka, W. Strupinski, M. Pokrywczynska, T. Drewa

**Affiliations:** 1 Chair of Urology, Department of Regenerative Medicine, Collegium Medicum, Nicolaus Copernicus University, Bydgoszcz, Poland; 2 Reconstructive Urology Working Group, Young Academic Urologists, European Association of Urology, Arnhem, Netherlands; 3 Faculty of Physics, Warsaw University of Technology, Warsaw, Poland; 4 Department of Biomedical Engineering, University of Science and Technology, Bydgoszcz, Poland; 5 Department of Biomedicine University Hospital, Basel, Switzerland; 6 Department of Histology and Embryology, Collegium Medicum, Nicolaus Copernicus University, Bydgoszcz, Poland; 7 Department of Clinical Pathomorphology, Collegium Medicum, Nicolaus Copernicus University, Bydgoszcz, Poland

**Keywords:** Bioinspired materials, Tissue engineering

## Abstract

Tissue engineering allows to combine biomaterials and seeded cells to experimentally replace urinary bladder wall. The normal bladder wall however, includes branched neuronal network propagating signals which regulate urine storage and voiding. In this study we introduced a novel biocomposite built from amniotic membrane (Am) and graphene which created interface between cells and external stimuli replacing neuronal network. Graphene layers were transferred without modifying Am surface. Applied method allowed to preserve the unique bioactive characteristic of Am. Tissue engineered constructs composed from biocomposite seeded with smooth muscle cells (SMC) derived from porcine detrusor and porcine urothelial cells (UC) were used to evaluate properties of developed biomaterial. The presence of graphene layer significantly increased electrical conductivity of biocomposite. UCs and SMCs showed an organized growth pattern on graphene covered surfaces. Electrical filed stimulation (EFS) applied *in vitro* led additionally to increased SMCs growth and linear arrangement. 3D printed chamber equipped with 3D printed graphene based electrodes was fabricated to deliver EFS and record pressure changes caused by contracting SMCs seeded biocomposite. Observed contractile response indicated on effective SMCs stimulation mediated by graphene layer which constituted efficient cell to biomaterial interface.

## Introduction

Regenerative medicine has developed over the last decades’ an interdisciplinary approach to urinary bladder regeneration that utilizes tissue engineering technology. Following this concept, a leading scenario aims to create a neobladder by combining different biomaterials with autologous urothelial and detrusor cells. These two components are expected to create a favourable environment for functional bladder wall regrowth. The biomaterial plays a pivotal role in this strategy as its biomechanical characteristic and bioactivity profile determine cellular events associated with regeneration^1^.

The urinary bladder is a temporary storage reservoir for urine. Both continence and voiding phases are active processes involving detrusor contraction or relaxation controlled by peripheral and central nervous system. In addition, passive mechanical properties of the bladder wall, such as viscoelastic force determine static and dynamic behavior of urinary bladder^2^. Within the bladder wall, there is multilayer branched neuronal network delivering and propagating signaling from the central nervous system (CNS) to the detrusor smooth muscle layer. Synchronous initiation of action potentials mediates the wavelike contraction, gradually embracing the whole bladder increasing intravesical pressure and generating urine outflow^3^.

To date, urinary bladder reconstruction with tissue engineering methods testing different usually acellular scaffolds has been used in 131 patients^4^. Nevertheless, none of them was successful in clinical translation due to the high complication rate and lack of long-lasting function. The major problem hampering advances in the field of urinary bladder tissue engineering is the simplification of the targeted neobladder structure in conducted research. Accordingly, one of the critical challenges hardly ever addressed in experimental settings was the restoration of the neuronal network within the neobladder wall^5^.

*De novo* regeneration of convolutional neural network within the neobladder hasn’t been ever tried due to lack of applicable technology. There might be however difrent ways to approach this dilemma. The alternative solution is to completely replace a native neural network with biocompatible current conductive material that might be linked to an external unit generating electrical stimulation^6^. However, before such a biocybernetics organ replacing unit may be created, there is a need to develop a biomaterial scaffold providing the interface between external stimuli and host tissue. Physiologically, the contraction of the detrusor muscle is initiated by meditators like acetylcholine and ATP (Adenosine Triphosphate) released from efferent nerve fibres. Subsequently triggered, discharge of the dispersing action potential is the direct signal contracting the smooth muscle layer^7^. On account of this two-stage mechanism, the chemical mediated stimulation could be replaced by direct electrical stimulation in the tissue-engineered bladder.

Inducing detrusor contraction by direct electrical stimulation is a concept which has been developed to initiate micturition reflex in patients with neurogenic bladder^8^. Research data derived from *in vitro* and *in vivo* models indicated that application of electrical stimuli directly to detrusor muscle may trigger effective contraction. Nevertheless the major challenge is to spread and sustain locally induced depolarization wave^9^. In comparison to heart urinary bladder doesn’t have recognized intramural conduction system delivering electrical stimuli to all bladder regions^10^. Creation of tissue engineered bladder offer possibility to develop *de novo* conduction system within bladder wall intended to distribute electrical stimulation aimed to trigger controlled contraction pattern of reconstructed smooth muscle layer.

In this study, we are proposing a new biocomposite biomaterial derived from the amniotic membrane (Am) covered with a graphene layer. The developed method of graphene transfer allowed to cover intact Am with solid graphene layers. The primary object was to obtain an electrical stimuli-responsive biomaterial tailored for provisional application in the tissue-engineered urinary bladder. The ability to deliver electrical stimuli opens a new perspective for the generation of dynamic tissue systems demanding a functional and responsive smooth muscle layer.

Am is used in urological tissue engineering due to its unique properties supporting the regeneration process^11^. Its excellent bioactivity profile is mediated by active molecules incorporated within the Am matrix. Am was applied for experimental reconstruction of the bladder wall in rat model and proved its ability to support regeneration of smooth muscle layer^12^. Furthermore, Am has universal capability to induce epithelization including urothelial layer regrowth^13^. Knowing this, the overriding assumption during interdisciplinary development of graphene transfer method was to avoid any modification of the Am structure impairing its biocompatibility profile. At any stage of biocomposite fabrication Am didn’t have any contact with cytotoxic, cross-linking or oxidizing substances.

Conductive biomaterials are of particular interests for tissue engineering, and recently many new biomaterials were introduced. Despite intensified research efforts, there are several significant limitations. The most substantial is reported to have reduced biocompatibility, and challenging necessity to combine hard or brittle conductive layer with softer and compliant natural derived biomaterial^14^. Therefore, an innovative strategy involving non-damaging graphene layer transfer on a biomaterial surface may overcome this limitation and become a novel solution worth further development and testing.

Graphene has emerged in the last decade as one of the most exciting nanomaterials whose electrical, mechanical, chemical, and structural properties can’t be obtained from either individual molecules or bulk materials^15^. In this context, the application of graphene in regenerative medicine enables to cross biological barriers which are inaccessible to more abundant commonly evaluated materials. The most crucial advantage of graphene is a neglectable influence on passive biomechanical characteristics of the tissue-engineered bladder wall. Therefore it doesn’t impair the ability to disdain during repetitive cycles of urine storage and micturition. Moreover, graphene, due to its nanostructure, might reach single effector cells analogously to healthy neurons and provide adequate interface replacing synapsis^16^. Graphene layers gradually overgrown with cells act as a nanoscale implant allowing to deliver electrical stimulation reestablishing cellular communication and regulating a cells’ behaviour or intrinsic features such as contraction^17^.

## Materials and method

### Biocomposite design

The sandwich-structured biocomposite material was constructed from frozen human Am and covered from the stromal side with two graphene layers. The stromal side was chosen due to its ultrastructure, which is irregular and offers a suitable scaffold for graphene attachment. Therefore the graphene layer could settle freely on pleated Am surface.

### Am preparation

Am was obtained according to the well-established protocol used in ophthalmology for Am transplantation Shortly, the donors was screened to exclude the risk of transmissible infectious diseases. The Am was obtained under sterile conditions after the elective caesarian section was washed out with an antibiotic solution, and the chorion was separated manually. With the epithelial surface up, Am was spread uniformly on nitrocellulose membranes. Am was then placed in a preservative medium and stored at −80 °C.

Am harvesting and experiment protocols were approved by Ethics Committee of Collegium Medicum, University of Nicolaus Copernicus (approval nr 045/17). All methods were carried out in accordance with national regulations on ethics and research in EU. Am was harvested from donors above 18 years old after obtaining written permission. All donors were informed consent for study participation.

### Graphene transfer

**Figure 1:**
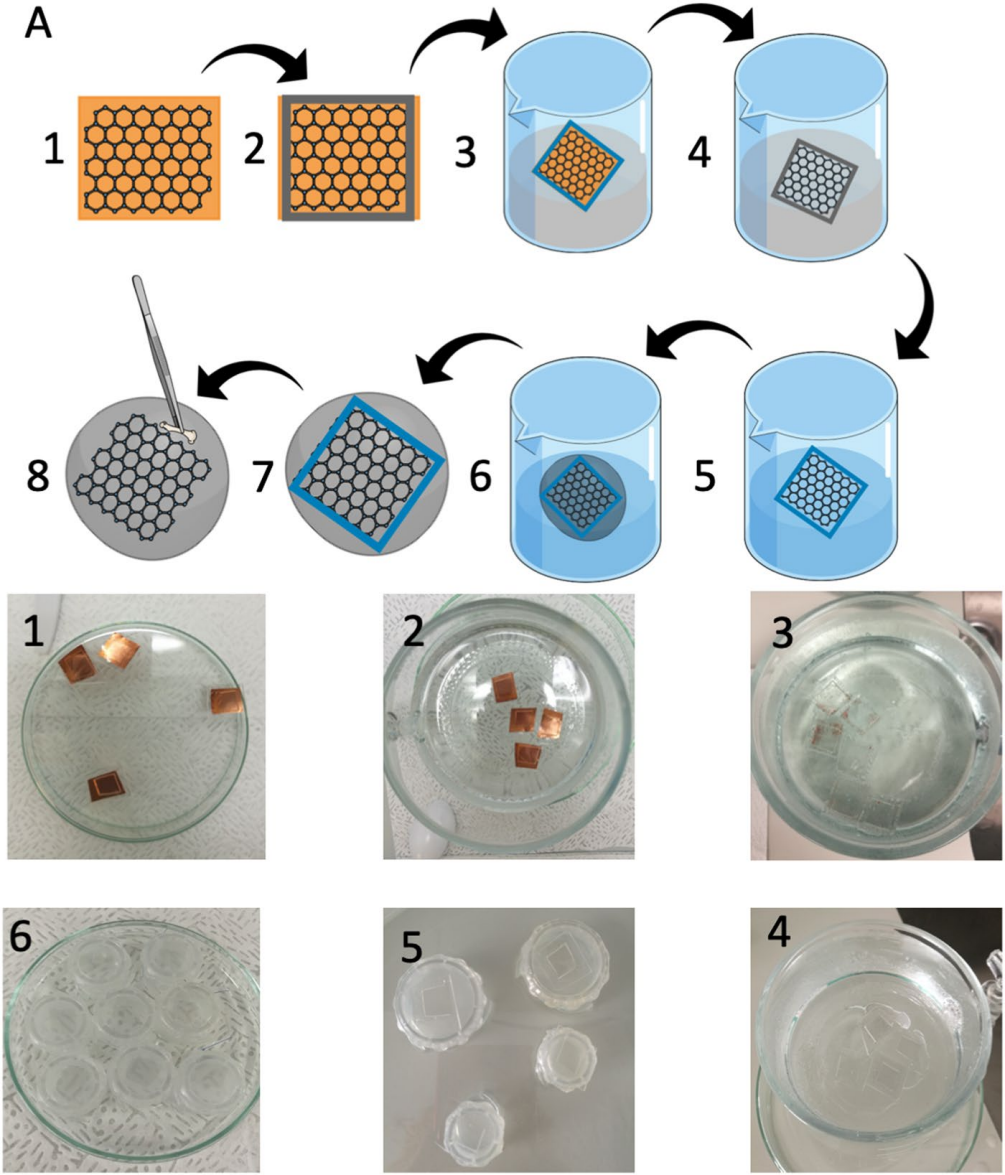
(**A**) Fabrication of biocomposite. (1) Graphene layer on copper foil. (2) PDMS frame adjusted to desired biomaterial shape. (3) Etching Cu foil by ammonium persulfate solution. (4) Floating graphene layer in PDMS frame. (5) Washing out ammonium persulfate. (6) Fishing graphene layer posited in PDMS frame with Am. (7) Graphene placed on Am surface. (8) Carful mechanical removing of PDMS frame.

Transferring graphene onto scaffolds required an individual approach to the transfer methods. Traditional PMMA (Polymethyl-methacrylate) approaches can’t be used since graphene’s adhesion to polymers is stronger than to extended surfaces, which Am is. As a result, graphene is removed from the substrate scaffolds during the dissolving of PMMA. To overcome this issue, we applied a modified marker-frame method (Fig. 1)^18^.

In this work, we used commercially available graphene (Graphenea Inc. USA) on 18 µm thick copper (Cu) foil. Since the marker-frame method is very demanding in terms of handling fragile graphene, at the very first step, we aimed at creating a bilayer of graphene on the copper substrate. We transferred the monolayer of graphene from the copper foil onto graphene on the copper foil by using electrochemical delamination methods^19,20^. Then a PDMS (Polydimethylsiloxane) frame instead of marker frame was positioned on a surface of bilayer graphene. Next, a sample was placed on the surface of an aqueous solution of ammonium persulfate (1 M) (Sigma-Aldrich, USA). After approximately 2 hours when Cu foil was completely etched, and bilayer graphene inside the PDMS frame floats on the surface of the solution, the sample was cleaned with a continuous and controlled flow of DI water. In the end, the bilayer graphene with a PDMS frame was carefully fished by Am and left at room temperature to dry and to improve both adhesion and contact between graphene and Am. Finally, the PDMS frame was mechanically removed from the Am, leaving the graphene layer alone on its surface.

### Analysis of the mechanical properties of the reconstructed bladder wall

Tensile tests were conducted on a load frame of a servohydraulic material testing machine (MTS 242.01 actuator, Eden Prairie, USA) as it was described previously^21^. All biocomposite samples (n = 5) included the same Am used as control (n = 5). The tested samples (10 mm length, 10 mm width) were mounted into flat grips with a gauge base of 10 mm. During the test, the specimen was longitudinally stretched at a rate of 0.2 mm/s until failure. The grip travel and specimen load were continuously measured over the test procedure with a precision force transducer (Interface, model 1500, measuring range 125 N, resolution 0.0625 N) and a MTS system linear variable differential transformer (measuring range 100 mm, resolution 0.01 mm). The Young’s elastic modulus (MPa) was estimated based on generated Stress/strain curves.

### Evaluation of electrical conductivity

**Figure 2:**
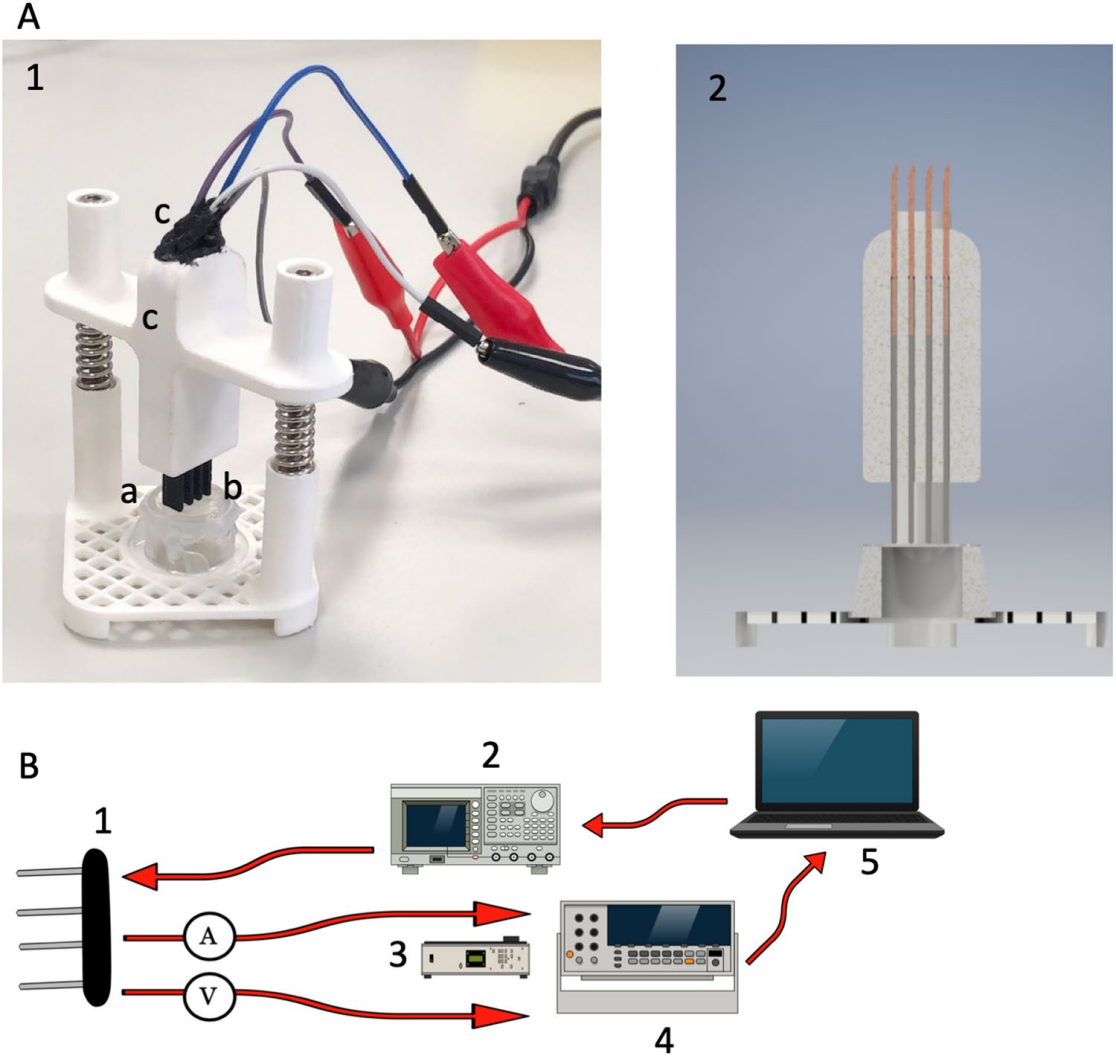
(**A**) PLA 3D printed device to conduct measurements. (1) Printed graphene based electrodes. (2) Regulated electrode grip allowing to precisely established contact points with material surface. (3) Silicon insulating connection between wires and electrodes. (4) Am fixed in Cellcrown. (**B**) Rendered model of constructed device showing electrode fixation and arrangement in cross view. (**C**) Setup used for conductivity evaluation. (1) Four-sensing probe. (2) Function Generator. (3) Multimeter. (4) Oscilloscope.

An in-house electrical stimulator was designed, and 3D printed using Polylactic acid (PLA) filament (Z-PLA Zotrax, Poland) (Fig. 2A,B) All of the materials used for the construction of this system are commercially available. The stimulator had modular construction to measure electrical conductivity and deliver stimulation during cell culture *in vitro*. It was designed also for Electrical Field Stimulation (EFS) of cells seeded on biomaterial fixed in CellCrown (Scaffdex, Finland). To our knowledge, there isn’t any commercially available testing system for this purpose. Electrical conductivity was measured using a four-sensing probe. The custom made probe utilized four 4 mm diameter electrodes printed from graphene PLA filament (0,6 Ω) (Graphene Supermarket, USA) spaced 2 mm apart. This gap was adjusted to graphene layer dimensions (10mmx10mm). Graphene containing filament was recently shown to be a suitable material for the fabrication of customized 3D printed electrodes^22^. The low-frequency (100 Hz) electrical conductivity at room-temperature was calculated for samples (n = 5) of each material. As a control, Am without a graphene layer and porcine bladder wall were utilized. The measurement setup consisted of the following devices: AFG3251 Arbitrary Function Generator (Tektronix, USA), DMM4050 multimeter (Tektronix, USA) and DMM4040 Osciloscope (Tektronix, USA). Raspberry Pi (Raspberry Pi Foundation, United Kingdom) computer gathered the data and calculated the conductivity of each material based on its electrical resistivity (Fig. 2C).

### SMC *in vitro* culture

Isolation and culture of bladder smooth muscle cells were carried out according to the protocol of Pokrywczyńska *et al*.^23^. Accordingly, urinary bladders from male domestic pigs were obtained from the local abattoir. All isolation and culture procedures were carried out in a laminar flow hood, using sterile techniques. The dissected smooth muscle layer of the urinary bladder was cut into small pieces and digested in collagenase II (0.15%, Gibco, USA) and dispase II (0.2%, Gibco, USA) solution (1.5 h, 37 °C). After digestion, the resulting suspension was filtered through 100μm nylon cell strainer (BD, USA) and centrifuged at 1500 × g for 5 min. Isolated cells were counted and seeded at a density of 2 × 10^4^ cells/cm^2^ in SmGM-2 culture medium (Lonza, USA). Bladder smooth muscle cells were cultured up to the third passage.

### UC *in vitro* culture

For isolation and culture of urothelial cells, we used the earlier optimized protocol of Pokrywczyńska *et al*.^24^. Accordingly, urothelial cells were isolated from porcine urinary bladders harvested from male domestic pigs during the planned economic slaughter in a local slaughterhouse. All isolation and culture procedures were carried out in a laminar flow hood, using sterile techniques. Bladder tissue pieces (1 × 1 cm) were incubated for 15 h at 4 °C in the conical tubes containing 10 mL dispase II (Gibco, USA) dissolved in the Hank’s Balanced Salt Solution (HBSS) (2.4 U/mL). After the dispase inactivation with an equal volume of DMEM/Ham’s F12 medium supplemented with FBS, the urothelial cells were carefully scraped with the blunt side of a scalpel, and the remaining tissue was discarded. Separated white conglomerates were transferred into a falcon tube and centrifuged at 1000 × g for 5 min. Isolated cells were counted using a trypan blue and seeded at a density of 4 × 10^4^ cells/cm^2^ in the commercially available CnT-57 growth medium (Cellntec, Switzerland). Urothelial cells were cultured up to the third passage in standard conditions at 37 °C with 5% CO2 atmosphere and 98% humidity until reaching 75–90% confluence.

### Graphene layer cytotoxicity

Glass coverslips covered with a graphene layer (n = 20) were applied to evaluate graphene cytotoxicity to SMCs. As a control group, coverslips without graphene were used (n = 20). SMCs were seeded (20 000 cells/cm^2^) and cultured until confluency was reached. The first stage of analysis included evaluation of cells morphology and growth pattern (Nikon Eclipse Ti-U, Japan). Then multi-staining (Calcein/Ethidium/DAPI)(LIVE/DEAD Viability/Cytotoxicity Kit, Thermofisher, USA) was used to determine the viability of cultivated cells on the graphene covered surface. The number of viable cells was estimated as follows. The number of ethidium positive cells was subtracted from the number of DAPI positive cells. Labeled cells were counted with a confocal microscope (Nikon Eclipse Ti-U, Japonia) within the circular ROI (Region of Interest) (n = 10). The analysis was performed at 40x magnification with particle analysis function in ImageJ (https://github.com/imagej/imagej).

Glass coverslips with a graphene layer (n = 5) were applied to evaluate SMCs adherence to surface covered with graphene. As a control group, coverslips without graphene were used (n = 5). SMCs were seeded (20 000 cells/cm^2^). Subsequently adherent cells were counted within ROI (n = 5) 3 h, 6 h and 12 h after seeding.

### Cell seeded constructs

For this research, two kinds of cell-seeded tissue-engineered constructs were created. The first one was composed of biocomposite and SMC, whereas the second one was built from biocomposite and urothelial cells. Am alone seeded with SMC or UC acted as a control. Scaffolds of both biomaterials were fixed in CellCrown12 inserts. Subsequently, 2,0 × 10^6^ SMCs and UCs were detached and seeded onto scaffolds’ surface in triplicate at 1 h intervals.

### Histological analysis of SMC seeded constructs

After six days of *in vitro* culture, specimens (n = 10) derived from SMCs seeded biocomposite grafts (n = 5) were fixed in 10% (v/v) neutral (pH = 7) buffered formalin and embedded in paraffin. Cross-sections of the entire area covered with graphene were prepared, and histological analysis using H&E staining was performed. Additional immunohistochemical staining using anti-SMA antibodies (R4A, Abcam, Great Brittan) and anti-smoothelin antibodies (Thermo Fisher Scientific, USA) was conducted to identify SMA and smoothelin cells and to distinguish them from the Am matrix. Stanning for smoothelin was conducted to verify whether *in vitro* cultured SMCs preserved their mature contractile phenotype. Digital images of anti-SMA and anti-smoothelin stained specimens were used for quantitative evaluation of SMC content. Immunohistochemistry Image Analysis Toolbox plugin (https://imagej.nih.gov/ij/plugins/) for ImageJ was applied to calculate SMA stained surface using generated histograms. The evaluation, according to this protocol, was done in square ROI (n = 10) for each specimen.

### Histological analysis of UC seeded constructs

After 7 days of *in vitro* culture, multi-staining (Calcein/Ethidium/DAPI) was used to determine viability and arrangement of UCs cultivated on biocomposite and Am surface. Labelled cells identified as alive were counted with a confocal microscope (Nikon Eclipse Ti-U, Japonia) by creating a square region-of-interest (ROI) (n = 10) at 40× magnification.

### *In vitro* electrical stimulation of SMC

**Figure 3:**
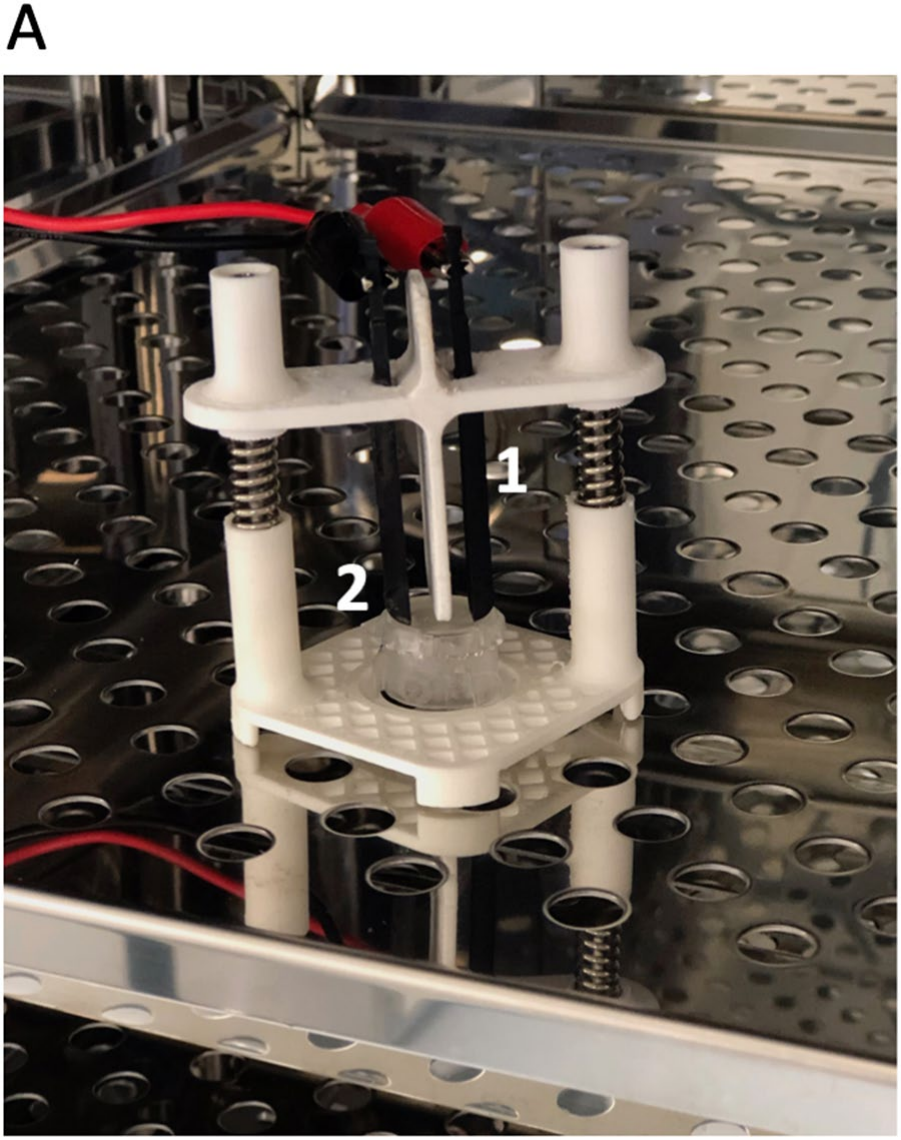
(**A**) 3D printed stimulator in CO2 incubator. (1) Graphene based electrodes (2) Biocomposite fixed in cellcrown.

Electrical stimulation of *in vitro* cultured SMC was conducted using an electrode printed from conductive graphene PLA filament (Graphene Supermarket, USA) (Fig. 3) The gap between the electrode (9 mm) was adopted to graphene layer dimensions (10mm × 10mm) to generate a stable electrical field. The stimulator was sterilized with ethylene oxide. Five biocomposite samples underwent evaluation. The control group consisted of Am seeded with SMC. After SMCs were seeded on biocomposite or Am and cultured for 72 hours, electric pulses with the constant amplitude were applied continuously in the incubator for 24 hours. The stimulation setup consisted of AFG3251 Arbitrary Function Generator (Tektronix, USA), DMM4050 multimeter (Tektronix, USA) and DMM4040 Oscilloscope (Tektronix, USA). The experiment was performed with an electric field of 3 V/cm. The strength of the electrical field was chosen based on available research discussing the impact of EFS on muscle cells *in vitro*^25,26^. Because of concerns about high thermal conductivity of graphene layer and potential increase of temperature induced by EFS, the biocomposite surface temperature was measured, 1 h after EFS initiation, with thermal camera (FLIR T530, USA). The measurement of the temperature was conducted 15 minutes after removing stimulator form cultivation incubator at room temperature. Following stimulation period cells were cultured an additional 48 hours. The multi-staining (Calcein/Ethidium/DAPI) was used to determine the impact of EFS on cell number and arrangement. Labelled cells identified as alive were counted with a confocal microscope (Nikon Eclipse Ti-U, Japonia) by creating a square (ROI) (n = 10) at 40× magnification. The orientation of cultivated SMC was established by a square ROI (80 × 80 pixels) (n = 10) analysis using ImageJ OrientationJ (EPFL, Switzerland) plugin. SMC bodies were manually outlined on DAPI/Calceine threshold images by fitting an ellipse to the selected region. The average cell orientation was determined by averaging all the values of deflection angles obtained from the ROI (n = 10).

### Response to electrical stimulation

**Figure 4:**
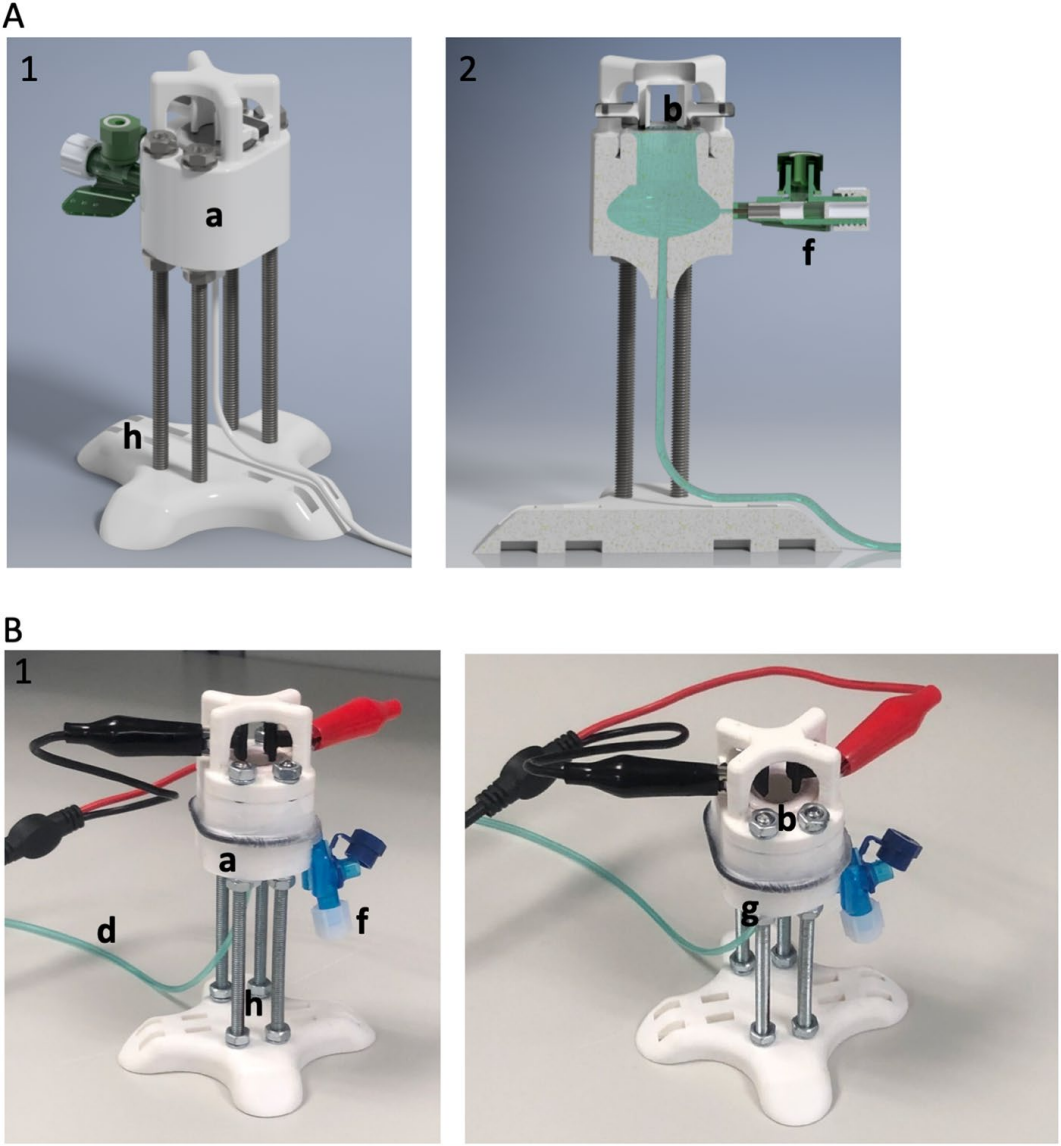
Rendered model of chamber constructed for recording activity of stimulated SMCs. (1) concept view. (2) the cross view. (**B**) PLA 3D chamber with attached electrodes and fluid-filled catheter. (a) Major chamber filed with saline solution. (b) Biocomposite placed and fixed with plastic ring. (c) Graphene based printed electrodes. (d) Urodynamic fluid-filled catheter fixed in the chamber. (f) Vent valve, made from PVC (Peripheral Venous Catheter). (g) O-ring sealed with parafilm. (h) Base platform with levelling screws.

We have designed an experimental setup to elicit false contractions from the cultivated muscle layer and record the increase of the pressure generated by the contracted graft (Fig. 4A,B). Each analysis was conducted on separate tissue engineered construct (n = 2). SMCs were cultivated 7 days on tested biocomposite scaffold and Am. To our knowledge, there isn’t any proved method to evaluate the contractions of tissue-engineered cell-seeded constructs and register its micromovements. The major concept of the applied strategy was to mimic urinary bladder behaviour. For this purpose, the oval construct corresponding to the shape of the urinary bladder was designed and 3D printed with PLA (Z-PLA Zotrax, Poland). The upper side was open and filled with fixed biomaterials that would act as a bladder doom. The fixing ring provided a sealed connection. On the bottom side, the scaled opening was placed to insert urodynamic fluid-filled catheter attached to a calibrated pressure transducer. The reservoir was filled with saline (5 ml) and vented through the valve. The cell-seeded side of the biomaterial was immersed in oxygenated Krebs solution (Sigma-Aldrich, USA) at 35 °C. To perform electrical stimulation, the customized printed graphene electrodes (0,6 Ω) (Graphene Supermarket, USA) were placed above. The adjustment of the electrode arrangement allowed to achieve optimal contact points with the biomaterial surface. SMCs responses were elicited by square wave pulses of EFS. Two different EFS patterns were applied: pulse duration 5 ms; 10 ms amplitude 7,5 V, 15 V, frequency 25 Hz, stimulation duration 4 s. Each analysis was conducted on separate tissue-engineered constructs (n = 2). Pressure changes were registered and recorded using a calibrated Duet urodynamic system (Mediwatch/Medtronic, USA).

### Statistics

Calculated results were reported as means with standard deviations. Statistical difference was evaluated using a one-way analysis of variance (ANOVA) with Tukey’s post hoc comparison at a significance level of 0.05.

## Results

### Characterization of Am based biocomposite

**Figure 5:**
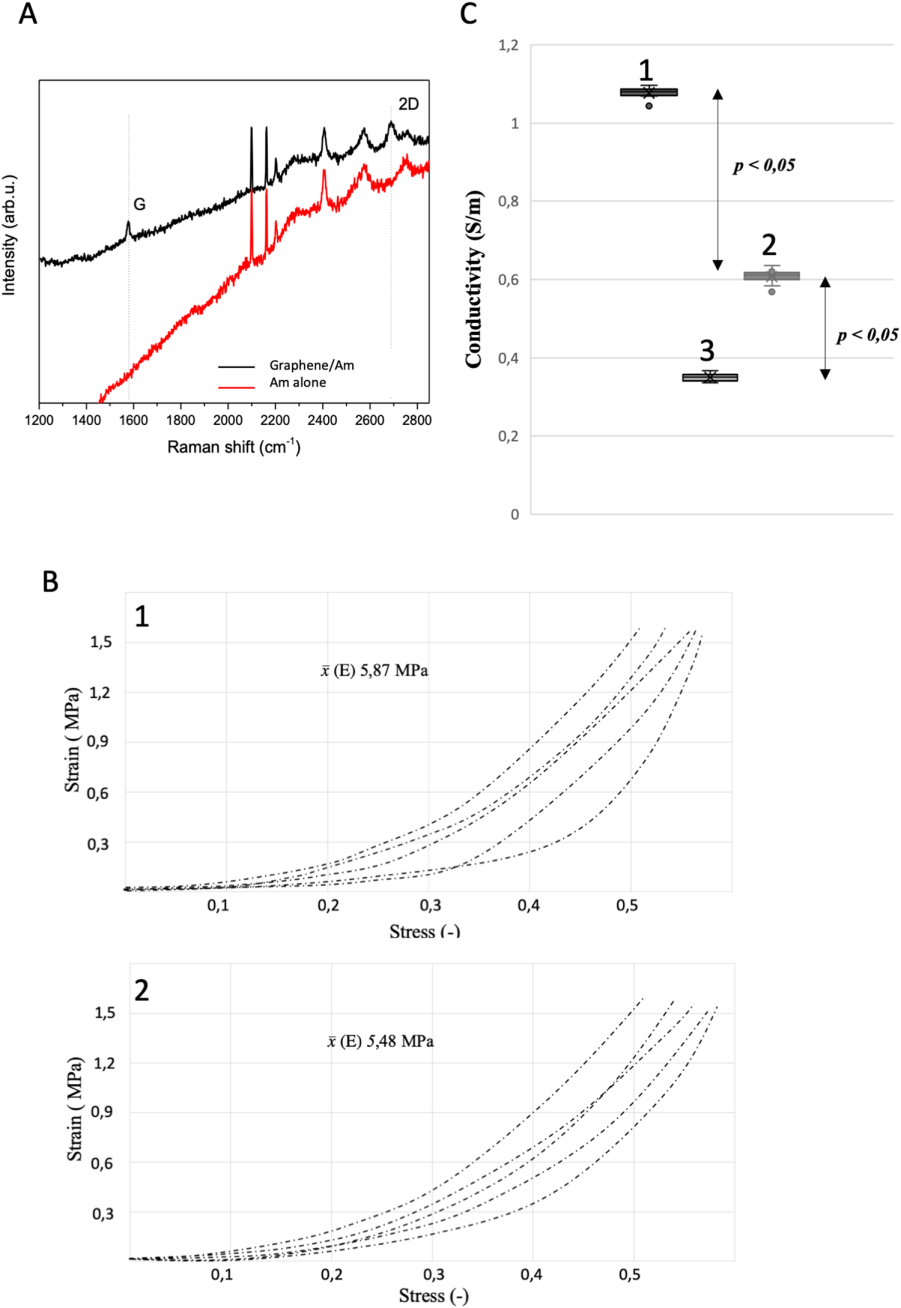
(**A**) Raman spectra of graphene on scaffold. (Black) and reference scaffold (red). Doted-lines indicate positions of G and 2D bands. (**B**) Measured electrical conductivity. (1) Biocomposite. (2) Porcine bladder wall. (3) Am. (**C**) The stress–strain curves represent the Young’s modulus of each tested sample. (1) Am. (2) Biocomposite.

The developed graphene transfer method enabled to cover the Am with a graphene layer without modification of the Am surface that could negatively impact its biocompatibility. The graphene layer was stably placed on the Am surface and didn’t tend to dislocate or to break during manual manipulation with the biocomposite. The characterization of the properties of the graphene transferred following the enhanced frame method was performed by Raman spectroscopy using a Renishaw system with a 514 nm laser line. The Raman spectra of graphene on the scaffold substrate (black line) and reference scaffold substrate (red line) are present (Fig. 5A). For the biocomposite spectrum, the two most prominent peaks in the Raman spectrum of graphene appear, i.e., the G band at ~1576 cm^−1^ and the 2D band at ~2688 cm^−1^, which confirms the presence of a graphene structure^27^. Evaluation of Young’s elastic modulus (E) indicated that either graphene layer itself or method of graphene transfer didn’t influence mechanical characteristic of Am which preserved its native elasticity (Fig. 5B). The manual handling of biocompoiste was indistinguishable from the regular Am during the processing and further testing.

### Biocomposite conductivity

Design printed stimulator allowed to precisely lower the electrodes to provide comprehensive and stable contact points. The calculated electrical conductivity of the graphene-based biocomposite, Am alone, and porcine urinary bladder was demonstrated in (Fig. 5C). The graphene layer significantly increased the Am electrical conductivity, which corresponded to the measured value in the standard bladder wall. Furthermore, obtained biocomposite conductivity values closely matched or even surpassed the native smooth muscle layer. Importantly, low standard deviations indicated a repeatable method of graphene transfer onto the Am surface and guaranteed reliable electrical properties.

### Graphene layer cytotoxicity

**Figure 6:**
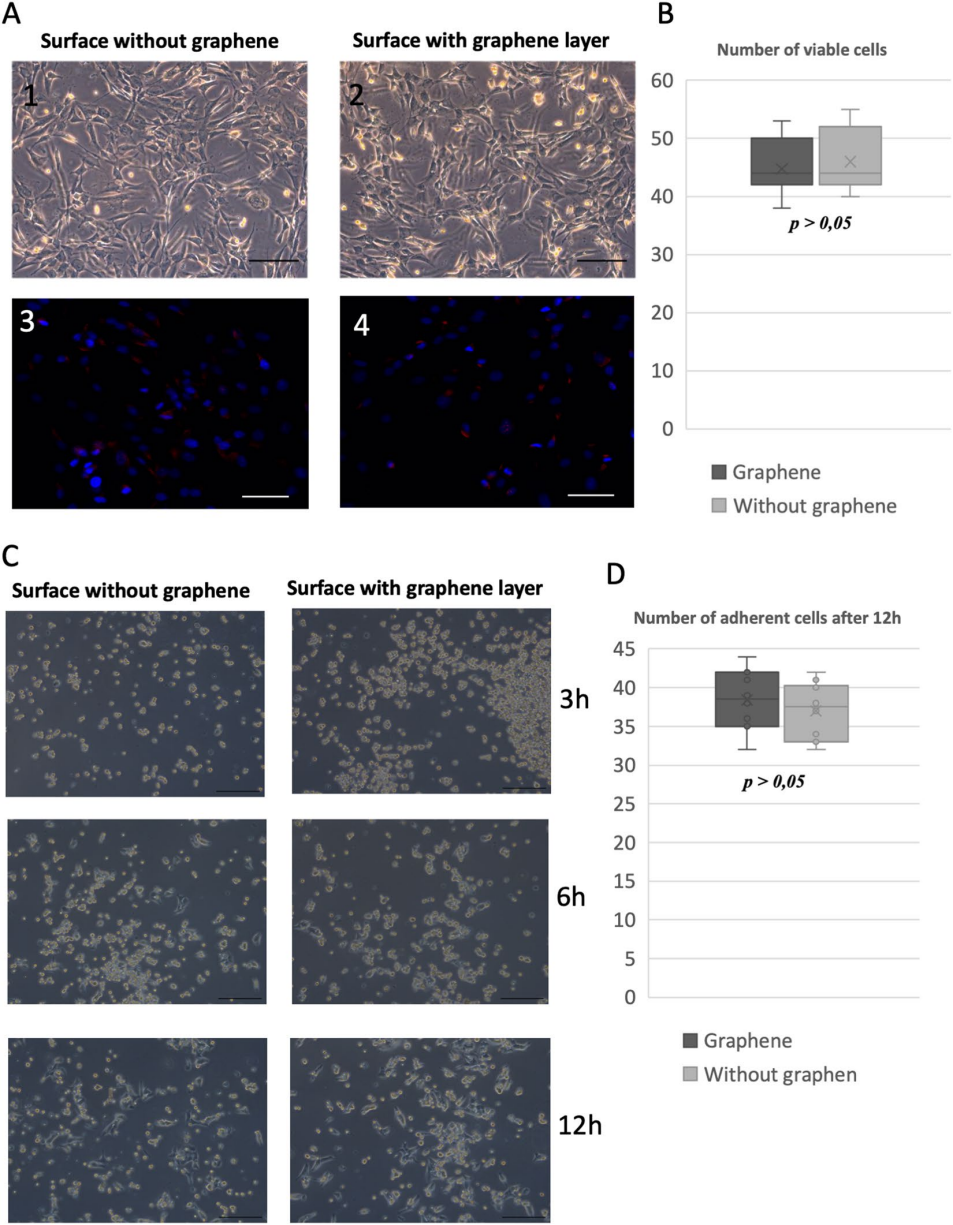
(**A**) Evaluation of graphene layer cytotoxity. (1) SMCs cultivated without graphene layer. (2) SMCs cultivated on graphene covered glass surface. (3) Live/Dead stanning, SMCs cultivated without graphene layer (DAPI/ethidium). (4) Live/Dead stanning, SMCs cultivated on graphene covered glass surface (DAPI/ethidium). Estimated number of viable SMCs cultivated on surface with/without graphene layer. Comparison of SMCs’ adherence to surface with/without graphene at different time points after seeding. (**A**) Estimated number of adherent SMCs cultivated on surface with/without graphene layer.

Due to the presence of the graphene layer, we didn’t notice an abnormal morphology of cultured SMCs. The cells were characterized by normal spindle shape, and the confluence of the cell populations was indistinguishable between groups. The graphene layer didn’t exert any cytotoxic effect or influence the SMCs adhesive properties. SMSs formed typical homogenous monolayer with single clusters. The number of cells cultivated on the graphene layer was comparable between groups, and the graphene layer didn’t decrease cell survival. The estimated cell number between groups is shown in Fig. 6(A,B). The graphene layer didn’t also impar cell adherence. We didn’t observed any differences in number of adherent cells after tested time periods (Fig. 6C).

### Histological analysis of SMCs seeded biocomposite

**Figure 7:**
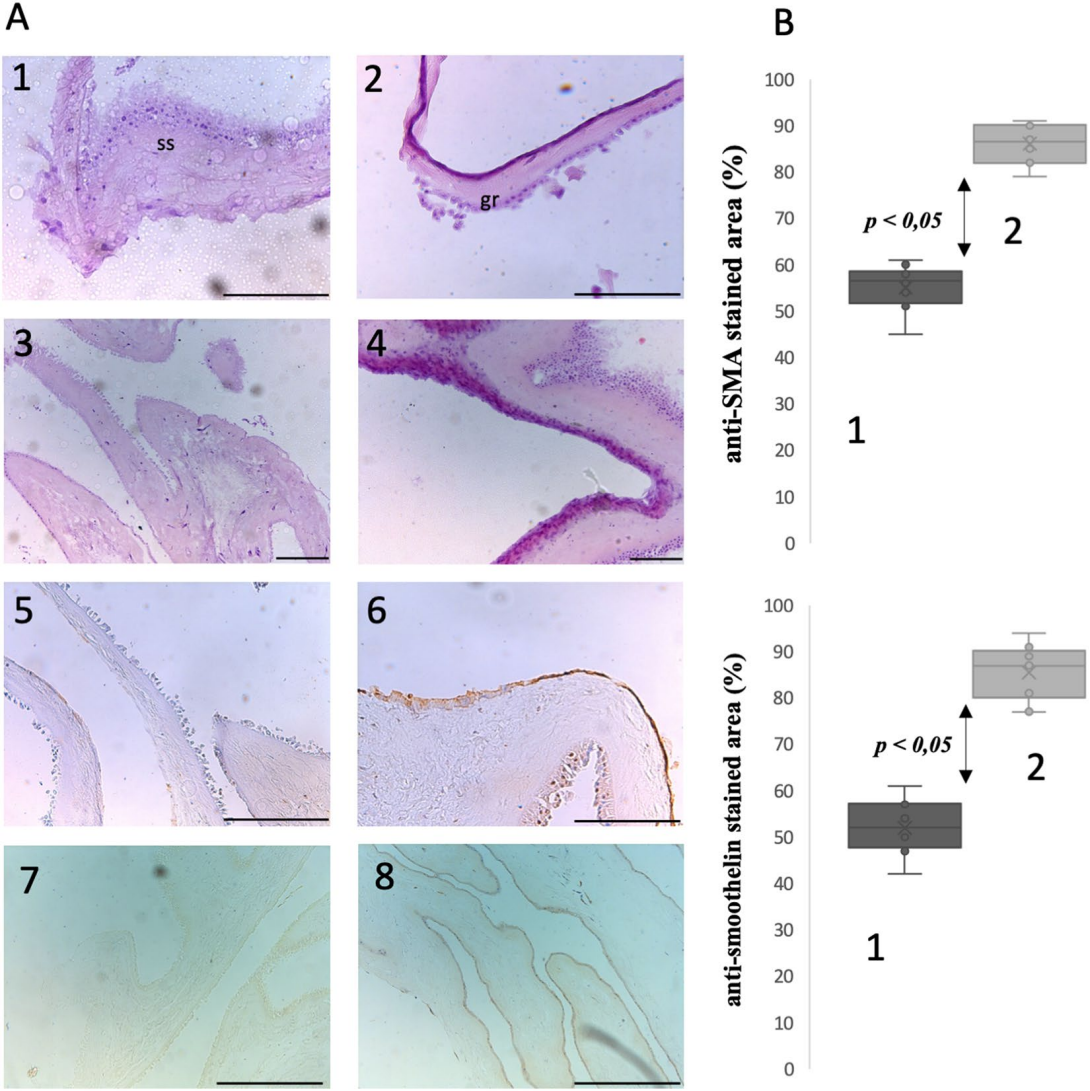
(**A**) H&E and SMA immunostaining of SMCs seeded biocomposite; all scale bars size - 200 µm. (1) Am alone, irregular texture of stromal side (ss) which was surface for graphene transfer (H&E). (2) Biocomposite, distinctive graphene layer adopt itself to natural Am folding (H&E). (3) SMCs seeded on stromal side of Am (H&E). (4) SMCs seeded on biocomposite, cells’ density is higher than on Am alone. Visible multilayer cell architecture (H&E). (5) SMCs seeded on stromal side of Am (anti-SMA immunostaining). (6) SMCs seeded on biocomposite, anti-SMA stained regions correlated with H&E stained areas rich in SMCs and revealed larger SMCs population then on Am alone. (7) SMCs seeded on stromal side of Am (anti-smoothelin immunostaining). (8) SMCs seeded on biocomposite, anti-smoothelin stained regions exhibited homogeneous layer with preserved mature contractile phenotype.B Quantified surface with positive (a) anti-SMA (b) anti-smoothelin immunostaining signal corresponding to SMCs. (1) Am. (2) Biocomposite.

In H&E stained specimens, the surface covered with graphene was recognized as sharply discriminated from the underlying Am (Fig. 7A). Nevertheless, the graphene layer seemed to attach without the tendency to separate or to fall off. In our opinion, the regions with graphene could be distinguished in H&E due to reduction of eosin by graphene oxide generated within the porous graphene mesh. Am with graphene formed an elastic layer without the tendency to break or tear (Fig. 7A). The graphene layer adjusted to the natural Am spatial configuration and natural surface deflection. Anti-SMA and anti-smoothelin staining revealed a homogeneous cell layer on the seeded surface indicating preservation of SMCs’ phenotype during *in vitro* cultivation. Graphene, as a hydrophobic carbon material didn’t impair the cells adhesive capability. Anti-SMA and anti-smoothelin staining were more intense on the biocomposite surface than Am alone (Fig. 7B). This observation may implicate that graphene created an optimal environment for SMCs growth and simultaneously allowed cells to maintain their primary mature phenotype.

### *In vitro* electrical stimulation of SMC

**Figure 8:**
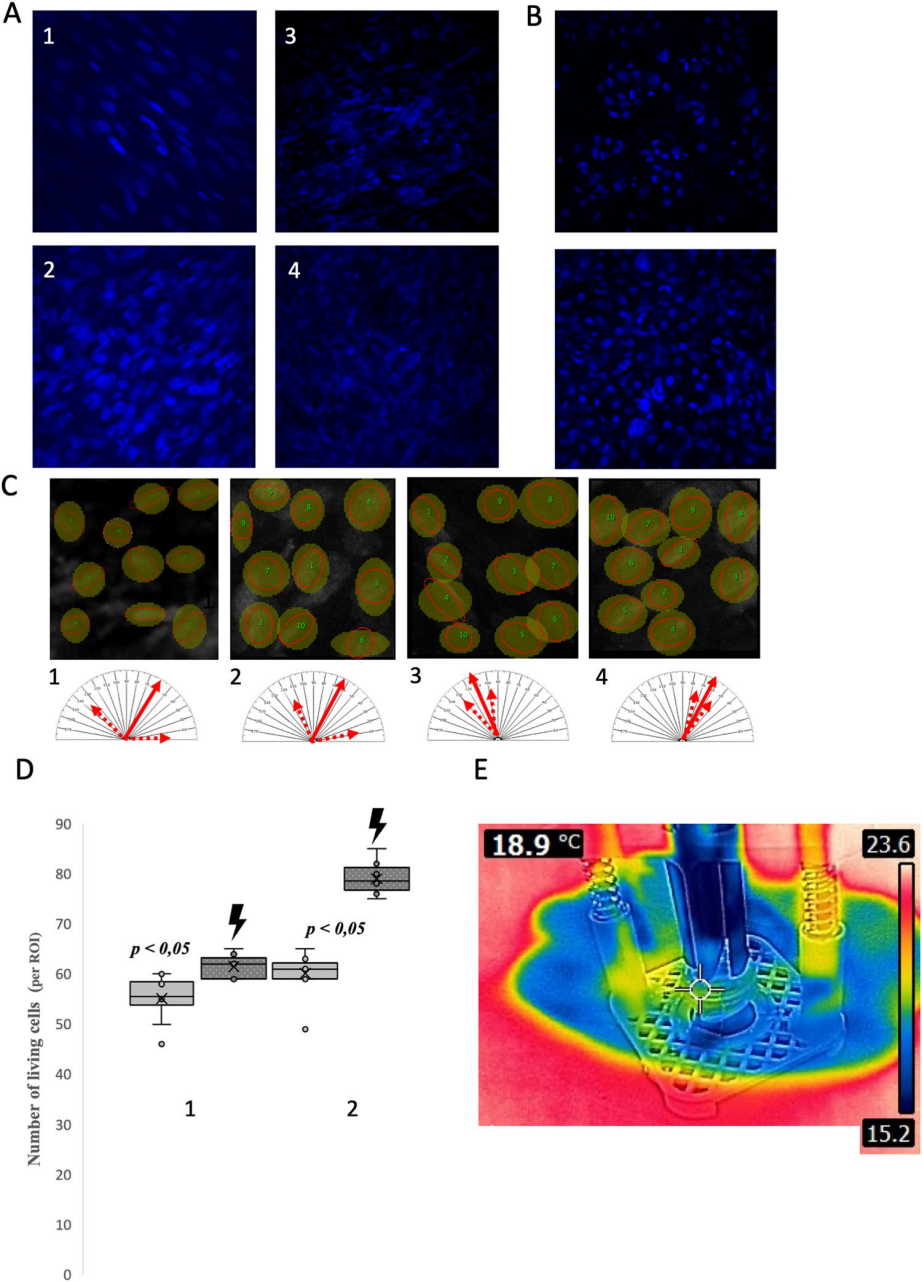
(**A**) Analysis of SMC growth on biocomposite and Am surface (DAPI, blue fluorescence),). (1) SMC on bicomposite before EFS, cells formed organized linear growth pattern. (2) SMC on bicomposite after EFS.Striking smoked glass effect visible is due to decreased transparency by graphene layer. (3) SMC on Am before EFS. (4) SMC on Am after EFS. (**B**) Analysis of UC growth on biocomposite and Am surface (DAPI, blue fluorescence), (1) UC on AM, cells form typical overlaying clusters. (2) UC on biocomposite, the graphene layer influenced arrangement and distribution of UCs. (**C**) Analysis of SMCs orientation (ImageJ); orientation of SMCs, average angular position is presented by filled arrow whereas minimal and maximal deviation by dotted arrows. (1) Am. (2) Am + EFS. (3) Biocomposite. (4) Biocomposite + EFS. (**D**) Number of cultivated SMCs before and after EFS (lighting). (1) Am. (2) Biocomposite. (**E**) Termovision image during *in vitro* EFS of SMCs. EFS didn’t increase temperature of biocomposite surface.

EFS didn’t increased temperature within biocomposite surface (Fig. 8E). Applied calcein/ethidium/DAPI staining revealed that the presence of the graphene layer organized the arrangement of SMCs. The SMCs population cultivated on the biocomposite surface were characterized with a noticeable regular linear organization in comparison to cells on the Am alone (Fig. 8A,C). EFS additionally improved the orientation of SMS cells with an exhibited tendency to form a linear pattern of growth with polarized cell orientation. Estimated cell orientation revealed a tendency to form homogenous localization pattern of SMC populations cultivated on the biocomposite. Graphene likely exerted a guiding effect modelling the spatial configuration of SMCs by providing stable adhesion points chosen by growing cells. The better adhesion properties of a tested biocomposite might also explain the higher number of SMC obtained during the cultivation period. The EFS led to a significant increase in the cell number on both the biocomposite and Am (Fig. 8D). Nevertheless, the increase in the SMC number was much higher in the case of the biocomposite. The cell population increased by almost a quarter. The graphene layer efficiently improved the passive electrical characteristics of the Am. The noticed elevation of SMCs implicated the achievement of effective interface mediated by graphene between the cellular component, biomaterial and external EFS. This guaranteed growing SMCs’ response to electrical stimulation.

### Histological analysis of UC seeded biocomposite

Confocal microscopy revealed that the organization of the urothelial layer exhibited an organized dispersed pattern on the biocomposite surface (Fig. 8B). Cells seemed to create mostly a monolayer with single clusters. The UCs’ arrangement might correspond to square like graphene network. We hypothesize that graphene layer might either guide growing UCs or alternately UCs might preferably adhere to surface covered with graphene layer. In the case of UCs grown on Am alone the distribution pattern was different. The cell clusters were more prominent with tendency to overlap and the regions between them weren’t rich in cells.

### Response to electrical stimulation

**Figure 9:**
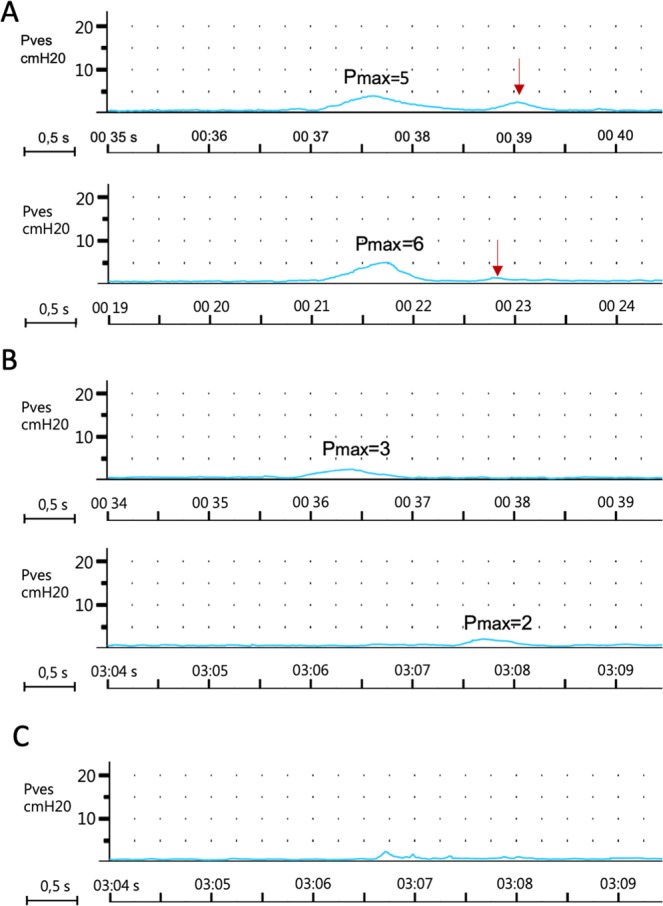
(**A**) Registered pressure changes inside 3D printed chamber mediated by SMC seeded biocomposite. Stimulation pattern: pulse duration 10 ms, amplitude 15 V. Red arrow indicates second spontanous wave of contarction. (**B**) Registered pressure changes inside 3D printed chamber mediated by SMC seeded biocomposite. Stimulation pattern: pulse duration 5 ms, amplitude 7,5 V. (**C**) The only registered pressure change inside 3D printed chamber mediated by Am alone. stimulation pattern: pulse duration 10 ms, amplitude 15 V.

The constructed device successfully delivered EFS to cells seeded on biomaterial scaffolds. EFS triggered contractions in tissue-engineered constructs and resulted in a temporary increase of pressure inside the testing device’s saline container. This is the first study documenting contraction of tissue-engineered constructs seeded with detrusor derived smooth muscle cells. The recorded contractions’ morphology corresponded to prolonged phasic detrusor muscle behaviour. The response to EFS was registered in all tested biocomposite samples (Fig. 9A–C). In contrast, in the case of Am alone, EFS managed to trigger a contraction response only at a single stimulation pattern (10 ms, amplitude 15 V). Moreover, the observed contraction was characterized by a low amplitude staccato shape. We interpreted this observation as a result of optimal electrical pulse propagation due to the presence of the graphene layer. Interfaced SMC with graphene was able to achieve a synchronous contraction-relaxation pattern and generate downward force. The highest pressure amplitude was registered after stimulation with 15 V. Interestingly, the duration of tonic contraction was similar in the two groups. It might be related to a similar number of the cellular components responsible for the generation of the contraction. Under this assumption, the EFS amplitude may be a factor determining the force of induced contraction. In the case of stimulation with 15 V, the spontaneous contraction wave was generated following the primary one. We can’t explain the mechanism of this phenomenon.

## Discussion

Regenerative medicine struggles to develop reliable tissue engineering technology for replacing diseased, inured or resected urinary bladders. Despite results indicating the feasibility of smooth muscles and urothelial layer regeneration, reestablishing bladder function emerged as a significant challenge which must be overcome to translate experimental settings into clinics^28^. The replacing of the neural network within the urinary bladder is “terra incognita” of regenerative medicine. This is the only study so far which raised awareness on this critical topic and demonstrated a feasible solution.

Since the development of graphene, this carbon material has been evaluated for application in tissue engineering. Graphene is used to design multifunctional biomaterials that can provide physical, electrical, and structural signalling to cells and tissues^29^. Graphene-based scaffolds were documented to support myogenesis and muscle cell proliferation^30^. In most of the reported cases, the applied approach utilized graphene oxide as a graphene source combined with hydrogels or electrospun biomaterials to endow them with electrical conductivity and cell instructive properties^31^. Notwithstanding this success, these materials primarily intended for soft tissue replacement weren’t adequate for urological tissue engineering due to insufficient mechanical resistance necessary to withstand the pressure of accumulating urine. The introduced graphene transfer technique enabling to create a biocomposite biomaterial from intact Am is a novel strategy. In contrast to mixing graphene particles with biomaterial components, we transferred pristine graphene layers onto the Am surface, which didn’t have contact with cytotoxic or cross-linking substances. Nonchemically-modified graphene is considered to be the most biocompatible form of graphene as it was shown not to alter neuron or muscle cell activity and simultaneously creates a favorable environment for electrical charge transfer^32^. As far as biocomposite structure is concern the next step should comprise evaluation of graphene layer structural coherency using Atomic Force Microspcopy (AFM). There is a need to analyze behavior of graphene layer in context of its elasticity and resistance for repeating load and unload cycles typical for urinary tract environment.

Am was extensively evaluated for urological tissue engineering, also in clinical settings^11^. An aqueous solution of ammonium persulfate is widely used for nanomaterial preparation. This agent, due to excellent solubility in water is easily washed away from the graphene layer. The concept of a stable graphene layer transfer is known in the electronics industry, but hitherto only metallic or polymeric materials acted as substrates^33^. This study introduced a new application for this technology in the field of biomaterials. The demonstrated method may be adopted to cover different biomaterials with graphene to fabricate conductive biohybrid materials that may find application in cardiologic or neurologic tissue engineering. The advantage of applied methodology for graphene transfer is the potential possibility to preserve intact Am bioactive content and mechanical properties. Nevertheless this assumption needs to be verified in further studies.

Graphene-based biomaterials were introduced as scaffolds supporting muscle cell proliferation. Most of them, however, were indented to promote myogenic differentiation and proliferation of striated muscle or cardiocytes^34,35^. Bladder detrusor has a unique histological structure with a syncytial ultrastructure divided into autonomous functional units connected by distributed innervation and a network of intestinal cells^36^. In contrast to striated muscle tissue, bladder’s smooth muscle syncytium creates a different environment for automatic propagation of action potentials^37^. From the electrophysiological point of view, local electrical stimulation tends to “scatter” in syncytium evoking action potentials with different inefficient shapes and sizes without a contractile response. In this situation, the external electrical pulse aimed to trigger an action potential must be simultaneously delivered at multiple points. Due to the excellent electrical properties of graphene, it offers the technological possibility to address this challenge by establishing an efficient biomaterial-cellular interface mediated coordinated response. In this context, graphene may become a milestone technical solution allowing to replace neuronal networks in artificial organs.

The graphene layer as a nanomaterial interacts with single cells promoting tight adhesion between cell membranes and the interfacing biomaterial^38^. We believe that the direct contact and exposure of SMCs to the electric charge was crucial to evoke an active contraction pattern. Moreover, graphene’s natural high flexibility prevented cells from detachment during construct manipulation and contraction itself. This graphene feature is particularly crucial for biomaterials indented to replace hollow organs that change volume during normal functioning such as the urinary bladder. The registered contractile activity of SMCs seeded on biocomposite was the result of graphene-supported stimulation propagation and a proper cell arrangement which enabled to generate stable force tensioning the Am. In comparison to cardiomyocytes electrophysiology of detrusor, SMCs is less known *in vitro*. Thus the applied stimulation parameters were chosen based on a few published studies concerning intact detrusor stimulation *in vitro*^39–41^. Parameters of electrical stimulation were chosen assuming that activation threshold of tissue engineered layer of SMCs should be similar to normal muscular layer of bladder wall.

There is a shortage of literature demonstrating contraction of tissue-engineered grafts *in vitro*, including the lack of a reference protocol. Kobayashi *et al*. determined that intestinal SMS seeded electrospun poly(3-caprolactone) scaffolds exhibited rhythmic contractions resulting in biomaterial contraction^42^. Krueger *et al*. reported the ability of muscle cells, forming myotubes seeded on graphene foam to mediate consolidated micromovements^43^. The designed, printed chamber, mimicking the bladder environment might become useful to evaluate the contraction ability of tissue-engineered grafts planned for reconstructive urology. We hypothesize that the fabricated chamber evaluating the tissue-engineered construct contractility based on pressure changes might be more sensitive in a tissue-engineered scenario than in stripe testing. It is challenging to obtain an SMCs seeded biomaterial with the ability to generate a singular vector contraction force recognized by a force transducer connected at both ends.

The role of modern biomaterials must include the ability to influence the cell arrangement to restore proper tissue architecture. The graphene layer passively organized the distribution of seeded SMCs and UCs. In the case of SMCs, it also directed cell orientation during EFS. From the beginning of research on the biotechnological application, the graphene ability to order cell arrangement is underlined^44^. The exact mechanism responsible for this phenomenon is unknown. Mainly, it is unclear whether graphene acts as a passive molecular pathway for proliferating cells or instead actively interacting with adhesive membrane molecules. Graphene incorporated with a collagen scaffold likely created micro-topographic structures that were preferably recognized by migrating neurons or muscle cells^45^. Moreover, being over one hundred-fold smaller than cells, graphene shouldn’t negatively affect the cytoarchitecture of the regenerating tissue contributing to the functionality of the reconstructed bladder wall.

From the perspective application in urology, a graphene-based biomaterial would also have additional advantages particularly important for clinical practice. The known antibacterial activity of graphene should prevent infection and facilitate healing of urinary tract wall after future reconstructive procedures^46^. Hydrophobic character of graphene may also reduce contact of regenerating tissue without developed urothelial barrier with urine. Urine has recognized cytotoxic activity which may impair regeneration^47^.

## Conclusions

The developed graphene based biocomposite successfully managed to deliver and to propagate external electrical stimulation to cultivated smooth muscle cells. The combination of conductive graphene and Am supporting cell growth resulted in creation of tissue engineered grafts capable of induced contraction *in vitro*. Further research are needed to evaluate behavior of this scaffold on *in vivo* models. Utilization of graphene for biomaterial design might become the first step to obtain biocybernetics platform aimed to control and regulate the function of tissue engineered bladder.
